# Preterm human milk at lactation weeks 1 and 4 categorized by maternal pre-pregnancy body mass index: Metabolomics and lipidomics datasets

**DOI:** 10.1016/j.dib.2020.106507

**Published:** 2020-11-06

**Authors:** Daniel T. Robinson, Lauren Balmert, Jami Josefson, Linda Van Horn

**Affiliations:** a Department of Pediatrics, Northwestern University Feinberg School of Medicine; Ann & Robert H. Lurie Children's Hospital of Chicago, Chicago, IL, USA; b Department of Preventive Medicine, Northwestern University Feinberg School of Medicine, Chicago, IL, USA

**Keywords:** Preterm, Human milk, Lactation, Pre-pregnancy body mass index, Overweight, Obesity, Metabolomics, Lipidomics

## Abstract

Human milk samples were prospectively obtained from women who delivered prior to the 32nd week of gestation [1]. The 36 preterm human milk samples analysed in this dataset were collected at week 1 and week 4 of lactation. Samples were categorized as being from women with normal pre-pregnancy body mass index (BMI 18–24.9 kg/m^2^) versus overweight/obese (BMI ≥25). Whole milk samples were frozen at −80 Celsius without prior processing and shipped for analysis on dry ice. Untargeted metabolomic and lipidomic platforms using UPLC-MS/MS and infusion-MS analysis for select lipids were performed by Metabolon. Lipidomic analysis included detection of complex lipids found in the milk fat globule membrane. Data were categorized by maternal BMI, week of lactation as well as gestational age at delivery. Data sheets are separated based on whether they report metabolomics versus lipidomics, as well as whether they report output from samples collected at week 1 versus week 4 of lactation. These data allow calculating relationships between clinical variables and human milk components. As an illustrative example, correlations between pre-pregnancy BMI and total milk fatty acids were calculated for this report using the Spearman correlation. These data will inform scientists of variability in milk composition attributable to maternal pre-pregnancy BMI as well as changes in milk composition as milk matures during lactation from week 1 to week 4. These data may best be used for generating hypotheses and justification of future work investigating whether maternal pre-pregnancy body mass index impacts preterm human milk composition.

## Specifications Table

**Table:** 

Subject	Perinatology, Pediatrics and Child Health
Specific subject area	These data provide measurement of preterm human milk composition according to maternal pre-pregnancy body mass index.
Type of data	Table Dataset/Spreadsheet
How data were acquired	Instruments: mass spectrometers, bioinformatics Instruments and software: Waters ACQUITY ultra-performance liquid chromatography (UPLC); Thermo Scientific Q-Exactive high resolution/accurate mass spectrometer; Orbitrap mass analyzer; Shimazdu LC with nano PEEK tubing; Sciex SelexIon-5500 QTRAP; a Laboratory Information Management System, Oracle 10.2.0.1 Enterprise Edition
Data format	Raw
Parameters for data collection	Untargeted metabolomics and lipidomics measured analytes in preterm human milk including complex lipids in the milk fat globule membrane. The 36 samples included met criteria for week of lactation (1 versus 4) and allow comparison of milk composition based on maternal pre-pregnancy body mass index.
Description of data collection	Women provided self-report of pre-pregnancy body mass index. Clinical data (e.g. gestational age at delivery) were abstracted from electronic medical records. Women expressed milk with an electric breast milk pump. Aliquots of milk were obtained from the entire expression after gentle swirling of milk and then frozen at -80 degrees Celsius prior to shipping to Metabolon on dry ice. No manipulation or processing of milk samples occurred prior to freezing and shipping. After metabolomics and lipidomics analysis by Metabolon, data output was provided in spreadsheet format.
Data source location	Institution: Northwestern University Feinberg School of Medicine City/Town/Region: Chicago, IL Country: USA
Data accessibility	With the article
Related research article	DT Robinson, HL Palac, V Baillif, E Van Goethem, M Dubourdeau, L Van Horn, CR Martin Long Chain Fatty Acids and Related Pro-Inflammatory, Specialized Pro-Resolving Lipid Mediators and Their Intermediates in Preterm Human Milk During the First Month of Lactation. Prostaglandins Leukot Essent Fatty Acids. 2017 Jun;121:1-6. Doi:10.1016/j.plefa.2017.05.003

## Value of the Data

**Fig. 1:**
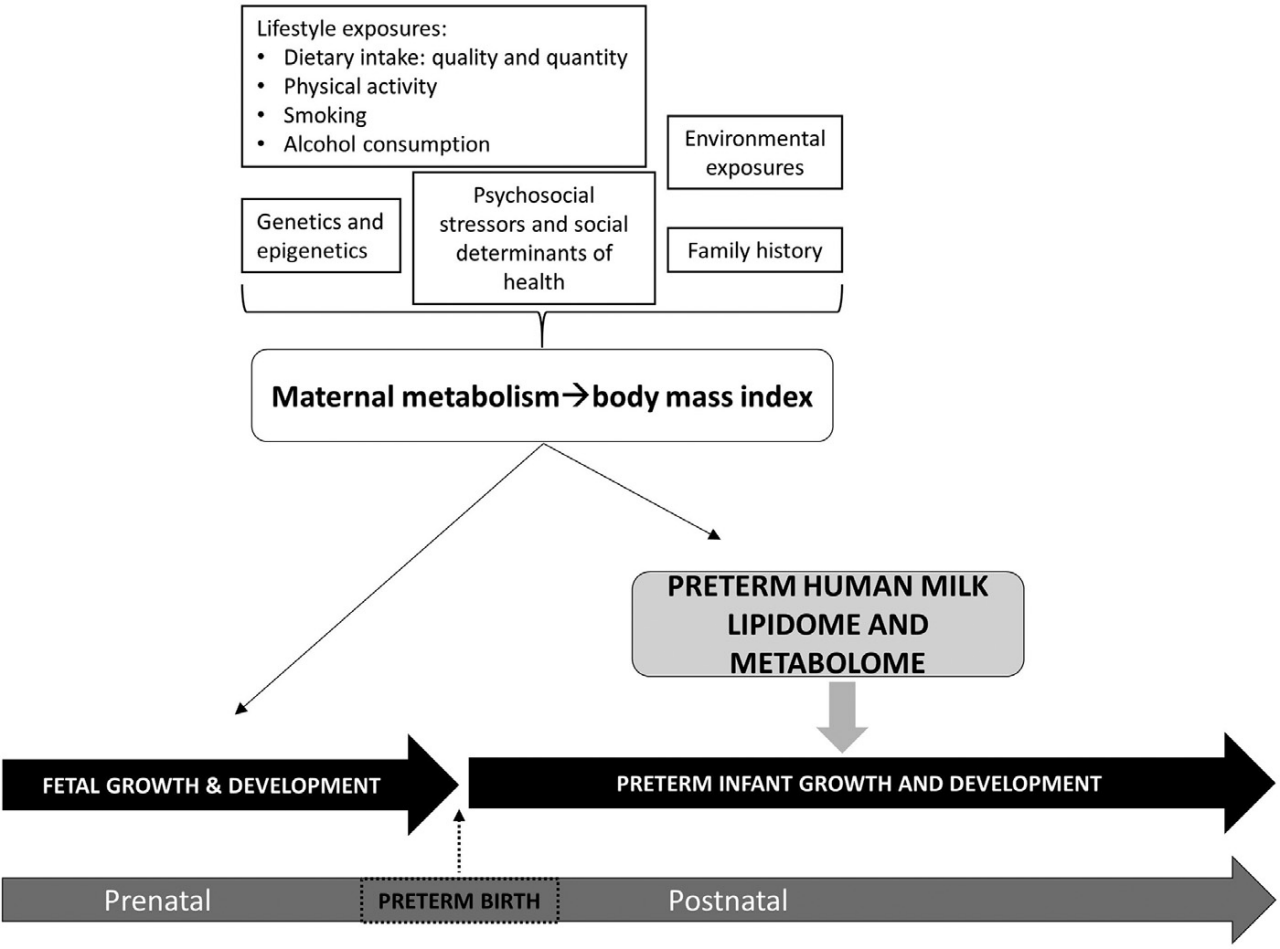
**Concept model defining relationships between maternal body mass index and preterm human milk lipidome and metabolome.** This model indicates that regulatory exposures on maternal metabolism and body mass index are multi-factorial. Maternal metabolism influences fetal growth and development during the prenatal time period. In the event of preterm birth, the maternal metabolism, reflected in body mass index, and its regulatory exposures influence preterm human milk composition as revealed in the milk metabolome and lipidome. Consequently, these alterations may influence preterm infant growth and development.

• Guidelines recommend human milk as the best form of nutrition for infants but limited information documents maternal influences, including pre-pregnancy BMI, on the known variability in milk composition.
• Pediatricians and nutrition scientists interested in variability and nutrient adequacy of human milk composition, including maternal influences on composition, can benefit from these data.
• Further investigation of the nutrient composition of human milk could identify the role of maternal pre-pregnancy body mass index and its association with preterm human milk composition [2], as well as general study of maternal diet quality and its influences on human milk composition [3].
• These data provide untargeted –omics analyses to inform scientists of variability in milk composition attributable to maternal pre-pregnancy BMI as well as changes in milk composition as milk matures during lactation from week 1 to week 4.
• Scientists may best use these data for generating hypotheses and justification of future work investigating whether maternal pre-pregnancy body mass index impacts preterm human milk composition and potential relevance to preterm infant health outcomes (Fig. 1).

## 1 Data Description

The data (Supplemental Data Set) contain untargeted lipidomic and metabolomic analyses of preterm human milk at weeks 1 and 4 of lactation, permitting not only cross-sectional analysis of milk composition but also longitudinal changes as human milk changes composition during lactation. The lipidomes provide data on 14 classes of lipids (e.g. phospholipids, sphingomyelin, tri- and diacylgylcerols, free fatty acids) as well as the fatty acid composition for each class. Total fatty acids in samples are also provided. Within these sheets, cells with no value indicate that the lipid was not detected and therefore not present in that sample. Metabolomic profiles provide analysis of 401 individually named metabolites reflecting metabolism in overarching categories including energy, amino acids, carbohydrates, nucleotides and vitamins. Data exist in one .xls file with 10 individual sheets, separated by lipidomic or metabolomic profiles (Table 1). Sheets are also separated by whether milk was expressed at week 1 or 4 of lactation. An additional excel sheet contains a legend defining the clinical variables. Each individual data set contains relevant clinical variables for consideration when evaluating influences on preterm human milk composition. This includes maternal perinatal health characteristics (e.g. pre-pregnancy BMI, age at delivery, race/ethnicity, primiparous status) as well as infant characteristics (e.g. gestational age at birth, birthweight, small for gestational age status). In considering the women with twin pregnancies, data for additional 5 infants is included (see Supplemental Data Set, sheet titled “Legend and Notes for Weeks 1 and 4”).

**Table 1:** Identification of lipidomic and metabolomic data sheets in supplemental data file.

Data sheet number	Title of data sheet	Contents
1	**Lipid Classes Week 1**	Concentrations of complex lipids in milk samples from week 1. Units are weight % (g/100g of total lipids).
2	**Lipid Classes Week 4**	Concentrations of complex lipids in milk samples from week 4. Units are weight % (g/100g of total lipids).
3	**Lipid Class uM Week 1**	Concentrations of complex lipids in milk samples from week 1 with units in micromolar.
4	**Lipid Class uM Week 4**	Concentrations of complex lipids in milk samples from week 4 with units in micromolar.
5	**Fatty Acid Composition Week 1**	Distributions of individual fatty acids amongst the complex lipids as well as total fatty acids in milk samples from week 1. Units are weight % (g/100g of total lipids).
6	**Fatty Acid Composition Week 4**	Distributions of individual fatty acids amongst the complex lipids as well as total fatty acids in milk samples from week 4. Units are weight % (g/100g of total lipids).
7	**Fatty acid uM Concentration W1**	Distributions of individual fatty acids amongst the complex lipids as well as total fatty acids in milk samples from week 1 with units in micromolar.
8	**Fatty acid uM Concentration W4**	Distributions of individual fatty acids amongst the complex lipids as well as total fatty acids in milk samples from week 4 with units in micromolar.
9	**Week 1 Metabolome**	Mass spectrometry data for individual metabolites in milk from week 1.
10	**Week 4 Metabolome**	Mass spectrometry data for individual metabolites in milk from week 4.
11	**Legend and Notes Weeks 1 and 4**	Key for the variables and complex lipid names as well as notes regarding twin pregnancies.

Within the 10 data sheets, the variable name is listed at the top of each column and each milk sample's data exists in a single row. Given the distribution of the 36 total samples amongst weeks 1 and 4 of lactation, sheets containing data from week 1 include output for 19 milk samples, and sheets displaying data for week 4 include output for 17 milk samples.

Illustrative analyses of data utility include associations between maternal pre-pregnancy body mass index (kg/m^2^) and total fatty acids in the preterm human milk samples collected at weeks 1 and 4 of lactation (Table 2). Associations were calculated using the Spearman correlation.

**Table 2:** Associations between maternal pre-pregnancy body mass index (kg/m^2^) and total fatty acids in preterm human milk. Associations were calculated using Spearman's correlations.

	Week 1 (*n* = 19)		Week 4 (*n* = 17)	
Fatty acid (wt%)	Spearman	*P*-value	Spearman	*P*-value
12:0*	−0.15439	0.53	0.12010	0.64
14:0	−0.15263	0.53	0.00980	0.97
14:1	0.17193	0.48	−0.53922	0.026
15:0	0.00351	0.99	−0.53431	0.027
16:0	0.11579	0.64	−0.24020	0.35
16:1	0.1	0.68	−0.15686	0.55
17:0	0.02982	0.9	−0.48284	0.0496
18:0	0.02807	0.91	−0.34804	0.17
18:1	0.30702	0.2	−0.23529	0.36
18:2	−0.2614	0.28	0.80147	0.0001
18:3	−0.18947	0.44	0.55147	0.022
18:4	−0.23051	0.34	−0.34903	0.17
20:0	0.26491	0.27	−0.36520	0.15
20:1	0.37018	0.12	−0.27100	0.29
20:2	0.43333	0.06	0.16422	0.53
20:3	0.34035	0.15	0.05882	0.82
20:4	0.3193	0.18	0.26225	0.31
20:5	−0.38246	0.11	−0.50735	0.038
22:0	0.08337	0.73	−0.36419	0.15
22:1	0.2807	0.24	−0.01961	0.94
22:2	0.41334	0.08	−0.24294	0.35
22:4	0.54912	0.015	0.24265	0.35
22:5	0.02105	0.93	−0.38381	0.13
22:6	−0.32632	0.17	−0.36520	0.15
24:0	0.25625	0.29	−0.00735	0.98
24:1	0.46424	0.045	−0.20356	0.43
26:0	0.16786	0.49	−0.25032	0.33
26:1	0.33925	0.15	−0.09872	0.71

⁎ Fatty acids reported as carbon chain length:number of unsaturated bonds.

## 2 Experimental Design, Materials and Methods

**Participant enrollment and sample collection:** Samples for this data set were obtained in a prospective, observational cohort of 30 women who delivered prior to the 32nd week of gestation [1]. Women were enrolled if they decided to express breast milk for their infant's feedings while infants were hospitalized in a level III neonatal intensive care unit in Chicago, IL, USA. Enrollment occurred during 2014–2015. Preterm human milk samples were collected throughout the first month of lactation, including at weeks 1 and 4 of lactation. Women provided pre-pregnancy body mass index (BMI, kg/m^2^) by self-report [4]. Self-reported weight in the pregnancy time period has shown accuracy when compared with medical records and has previously been utilized in NIH-funded trials [5], [6], [7].

Preterm human milk was collected from participating women after they completed a full expression with an electric breast pump. From the expression, 1–2 mL aliquots were obtained and frozen at −80 °C. The whole milk samples were stored without processing prior to freezing. During consent for participation in the original cohort study, participants selected whether to opt-in for future studies on human milk with residual specimens. Samples for this dataset were shipped on dry ice to Metabolon for analysis. No delays, mishandling or unexpected thawing occurred during shipping and receipt of samples.

Thirty six residual samples for this study were selected from those collected at weeks 1 and 4. With a priority to select samples from women with no pregnancy-associated morbidities, we then categorized samples based on maternal pre-pregnancy BMI in to 2 groups: normal (BMI 18 to <25) versus overweight/obese (BMI ≥ 25). For week 1, a total of 19 human milk samples were included (9 from women with normal pre-pregnancy BMI, 10 from women with pre-pregnancy overweight/obese BMI). For week 4, a total of 17 human milk samples were included (8 from women with normal pre-pregnancy BMI, 9 from women with pre-pregnancy overweight/obese BMI).

**Human milk sample preparation:** Protein precipitation occurred using methanol for extraction after recovery standards were added to samples. Organic solvents were removed using a TurboVap® and then extracts were stored under nitrogen overnight.

**Sample quality assurance and quality control:** Both experimental samples and controls (pooled matrix sample for technical replicate; extracted water for blanks; QC standards in analyzed samples) were run for monitoring instrument and chromatography performance. Variability of instruments was assessed using median relative standard deviations of the standards.

**Ultrahigh Performance Liquid Chromatography-Tandem Mass Spectroscopy (UPLC-MS/MS):** Per Metabolon standards, all analyses used: 1. Waters ACQUITY ultra-performance liquid chromatography (UPLC), 2. a Thermo Scientific Q-Exactive high resolution/accurate mass spectrometer, 3. MS interfaced with a heated electrospray ionization (HESI-II) source, and 4. Orbitrap mass analyzer at 35,000 mass resolution. Extracts were dried and then reconstituted. Standards were added to reconstituted extracts. Conditions were optimized for chromatography to detect hydrophobic as well as hydrophilic compounds. Considering all optimization methods, the total range scanned covered 70–1000 m/z.

**Analysis of Complex Lipids:** Lipid extraction used methanol:dichloromethane in conjunction with internal standards. Nitrogen was used to concentrate extracts which were then reconstituted in ammonium acetate dichloromethane:methanol (50:50). Infusion-MS analysis was completed on a Shimazdu LC using nano PEEK tubing in conjunction with Sciex SelexIon-5500 QTRAP which used MRM mode for a total >1100 MRMs. Quantifying lipid species used peak area ratios of both target compounds as well as internal standards with internal standard concentrations. Concentrations of lipid classes were calculated from the sum total of all species in a class. To calculate fatty acid composition, each class was evaluated to identify the proportion of that class comprised by an individual fatty acid.

**Bioinformatics:** Four constituents contributed to bioinformatics processing: a Library information management system, software for data extraction and peak-identification, data processing for compound identification as well as data analyst time for interpretation.

**Data extraction for compound identification:** Peak identification involved comparisons to a Metabolon library that included purified standards; unknown entities that recurred were also identified. Relevant library information for identification included: retention time/index, mass to charge ratio (m/z), chromatographic data for catalogued molecules. This also includes MS/MS spectral data.

**Curation:** QC and curation procedures optimized removing system artifacts, inappropriate assignments as well as background noise in order to assure consistent, accurate identification of compounds. This was facilitated by Metabolon's proprietary software.

**Metabolite quantification and data normalization:** Metabolites were quantified using area under the curve for peaks. When necessary, and only if specimens were run over multiple days, data were normalized to a median of 1.

## Ethics Statement

The authors have adhered to all standards for reporting original research as delineated in the following sections:

### Protection of human subjects

This work described has been carried out in accordance with the Code of Ethics of the World Medical Association (Declaration of Helsinki) for experiments involving humans. All study procedures were approved the local institutional review board and informed consent was obtained prior to any study procedures involving human subjects (IRB approval #2018-2037/84344).

## CRediT Author Statement

Author roles are:

**Daniel Robinson:** Conceptualization, Study Methodology, Investigation, Data Curation, Writing- Original draft, Project administration, Funding acquisition; **Lauren Balmert**: Formal analysis, Data Curation, Writing- Review & Editing, Visualization; **Jami Josefson**: Conceptualization, Study Methodology, Writing- Review & Editing; **Linda Van Horn**: Conceptualization, Study Methodology, Writing- Review & Editing, Supervision.

## Declaration of Competing Interest

None.

## Appendix Supplementary materials

Image, application 1
